# African American Adolescents and Young Adults, New Media, and Sexual Health: Scoping Review

**DOI:** 10.2196/19459

**Published:** 2020-10-05

**Authors:** Sierra Teadt, Jade C Burns, Tiffany M Montgomery, Lynae Darbes

**Affiliations:** 1 School of Public Health University of Michigan Ann Arbor, MI United States; 2 Department of Health Behavior and Biological Sciences School of Nursing University of Michigan Ann Arbor, MI United States; 3 College of Nursing & Health Professions Drexel University Philadelphia, PA United States

**Keywords:** African American, adolescent, young adult, technology, safe sex, sexually transmitted infections, sexual behavior, new media, social media, internet

## Abstract

**Background:**

Rates of sexually transmitted infections and unintended pregnancies are disproportionately high among African American adolescents and young adults (AYA). New media platforms such as social networking sites, microblogs, online video sites, and mobile phone applications may be a promising approach in promoting safe sex and preventing sexually transmitted infections.

**Objective:**

The purpose of this scoping review was to address promising approaches in new media that may serve as valuable tools in health promotion, prevention, education, and intervention development aimed at African American AYA.

**Methods:**

An electronic search was conducted using Google Scholar, Scopus, Cumulative Index to Nursing and Allied Health (CINHAL), and PubMed online databases. Concept blocks and MeSH terminology were used to identify articles around African American youth and new media.

**Results:**

The search yielded 1169 articles, and 16 publications met the criteria. Studies from the review found themes in new media that included feasibility, changing attitudes, and improving knowledge related to sexual health behavior among youth of color.

**Conclusions:**

New media is a promising and feasible platform for improving the sexual health of African American AYA. Further research is suggested to better understand the benefits of new media as a sexual health promotion tool among this specific population.

## Introduction

Rates of sexually transmitted infections (STIs) and unintended pregnancies are disproportionately high among African American adolescents and young adults [1]. Multiple personal and social factors contribute to this increased risk, including (1) having more sex partners than youth of other ethnicities, (2) early sexual debut, (3) inconsistent condom use, and (4) limited access to sexual health promotion resources [2-5]. STI prevalence and other risk factors among this population are particularly acute in minority communities of low socioeconomic status [6].

While leading public health and medical organizations support comprehensive sex education for adolescents and young adults, there are several limitations of traditional or “formal” sex education platforms that often take place in structured settings (eg, schools, youth centers) [7]. According to the Centers for Disease Control and Prevention (CDC), sexual health education is more commonly required in high school than middle or elementary school [7]. As a result, this information is provided too late for many African American adolescents, as it is estimated that 20% of youth have reported having had sex by the age of 15 years, and it is known that African American youth report age at first sexual intercourse earlier than their peers [8]. Additionally, most schools fail to provide instruction covering all 16 sexual health topics that the CDC considers essential; less than half of high schools and only 20% of middle schools cover all 16 topics [7]. Furthermore, only 35% of high school students and only 10% of middle school students were taught how to correctly use a condom in 2014 [7]. Lastly, 88% of schools in the United States allowed parents to exempt their children from sexual health education in 2014 [7].

New platforms for sexual health promotion and prevention aimed at African American adolescents and young adults are especially important, as this population faces multiple barriers to active engagement in health care [9]. Such barriers include the inability to pay for health care services, lack of transportation, long waiting times, conflict with work or school schedules, confidentiality concerns, and embarrassment that is attached to sexual health services [10,11]. Additional barriers include actual or perceived fear and distrust of health care institutions, discrimination, and provider bias [11]. Additionally, minority youth in urban settings report prioritizing basic needs, such as housing, food, and transportation, over HIV risk reduction or prevention [1]. These individuals often experience difficulty when navigating and affording quality sexual health services [12]. Similarly, minority youth have higher rates of medical poverty compared to their white counterparts [1]. The financial, cultural, and institutional barriers to health care among minority youth often result in a lack of access to comprehensive adolescent health services, including sexual health services [1].

One promising approach to improving adolescents’ and young adults’ high-risk sexual behavior and filling critical gaps in knowledge due to formal sex education platforms may be to promote safe sex and STI prevention via new media [13]. These platforms include social networking sites, microblogs, online video sites, and mobile phone applications (see Table 1) [13]. African American adolescents not only use cell phones and the internet at high rates but they are also open to seeking and receiving sexual health information via new media due to its accessibility and ability to provide a wide range of information [14]. While African American adolescents and young adults are actively using new media on a day-to-day basis, it is essential to assess the ways these platforms have been — and could be — leveraged to promote engagement in health care and reduce risk-related behaviors [15].

**Table 1:** Classification of new media.

Types of new media^a^	Primary purpose	Examples
Social networking sites	Peer networking	Facebook, Instagram, MySpace
Collaborative websites	Information sharing, discussion	Wikipedia, AskFM, answer.com
Blogs (and microblogs)	Opinion sharing, discussion	Twitter, Tumblr, Blogger
Content communities	Entertainment, information sharing	YouTube, Snapchat, Reddit
Virtual reality/online gaming	Simulate experiences, entertainment	It's Your Game: Keep it Real, PlayForward
Communication/messengers	Discussion	WhatsApp, Facebook Messenger, GroupMe

^a^Not inclusive of all new media platforms that exist today. Adapted from [16].

Smartphone use and engagement with social media are high among African American adolescents and young adults [17]. They are already being used by this age group to search for general health information and sensitive health topics [17]. New media platforms provide a venue for greater anonymity and client sensitivity, which are critical to an adolescent’s self-expression [18,19]. As the use of new media continues to increase among adolescents and young adults, these platforms may serve as valuable tools in health promotion, prevention, education, and intervention development aimed at this population.

Many evidence-based sexual risk-reduction interventions target African American youth. For instance, the “Compendium of Evidence-Based Interventions and Best Practices for HIV Prevention” is a collection of good and best-evidence sexual risk-reduction interventions compiled by the CDC [20]. This collection includes 59 evidence-based interventions, 10 of which specifically target African Americans and 3 of which target African American youth. None of these interventions include new media as a method for intervention delivery. So, while the extent of the sexual health risk among African American adolescents is evident, it is not clear which health promotion strategies (eg, peer-to-peer vs online) will be most effective in reducing risky sexual behaviors among this population [21].

Accordingly, the goal of this review was to investigate the various forms of new media, the current state of evidence on the platforms used, and how it can improve health care engagement and sexual health outcomes among African American youth**.** The following research question is addressed: How is new media useful in the delivery of STI prevention and risk-reduction interventions among African American youth?

## Methods

### Search Strategy

A scoping review methodology was selected as it (1) helped to identify review parameters, (2) identified a process of mapping the existing literature, and (3) explored a research gap [22,23]. We used the framework by Arksey and O’Malley [22] along with the Preferred Reporting Items for Systematic Reviews and Meta-Analyses (PRISMA) guidelines [23,24] for this review. An informationist was consulted to provide expertise in creating tailored search strategies on this topic. The literature search occurred between March 2018 and December 2018 using the following electronic search engines and databases: Google Scholar, Scopus, Cumulative Index to Nursing and Allied Health (CINHAL), and PubMed. Concept blocks were used in each search engine to combine keywords in the title and abstract and used with MeSH terminology. A manual review of systematic reviews was also employed. See Table 2 for a list of standard terms used. A name search was then conducted on articles from technology and media experts along with a manual review of relevant articles and a CDC list of evidence-based interventions around youth and sexual health. Finally, a table of evidence was constructed to organize the articles by the level of evidence, sample size, type of new media used, results, and any limitations within the study.

**Table 2:** Search strategy concept blocks.

Concept block	MeSH terms	Title and abstract terms	Additional terms
Adolescent	Adolescent, young adult	Adolescent, youth, young people, young adult	Teenager, teen
New media	Social media, cell phone, internet, telemedicine, text messaging, multimedia, mobile applications, smartphone	Social media, new digital media, internet, mobile phones, text messaging, Facebook, instant messaging, multimedia, online social networks, computer, technology, mobile health, smartphone, Web 2.0, eHealth, mHealth^a^, SMS	Apps, SNS^b^, social networking sites
Sexual health	Risk reduction behavior, safe sex, sexually transmitted diseases, condoms, HIV infections, sex education, sexual behavior	Risk reduction, sexual health, HIV/STI^c^ risk, HIV prevention, sexually transmitted diseases, sexual practices, sexually transmitted infection	N/A^d^
African American	African American	African American	Black, minority group

^a^mHealth: mobile health.

^b^SNS: social networking site.

^c^STI: sexually transmitted infection.

^d^N/A: not applicable.

### Selection and Assessment of Articles

An initial search trail document was created to detail all review findings. Duplicates were screened for independently and then by team review. Next, abstracts were reviewed for relevance, followed by a full-text review. Discrepancies related to the retention or removal of articles were discussed until consensus was achieved. Finally, themes were created from the existing domains. Inclusion criteria included (1) African American adolescents and young adult participants aged 13-24 years, (2) sexual health, (3) new media use as defined in our background section, (4) publication after 2009, and (5) publication within the United States. Study samples were required to reflect the US African American population (13%) [25]. Exclusion criteria included (1) non-English language articles; (2) letters to the editor, opinions, commentaries, and narrative reviews; (3) studies used for recruitment only; and (4) text messaging, which has been well-covered within the literature for the adolescent and young adult population.

## Results

### Descriptive Characteristics of Reviewed Articles

**Figure 1:**
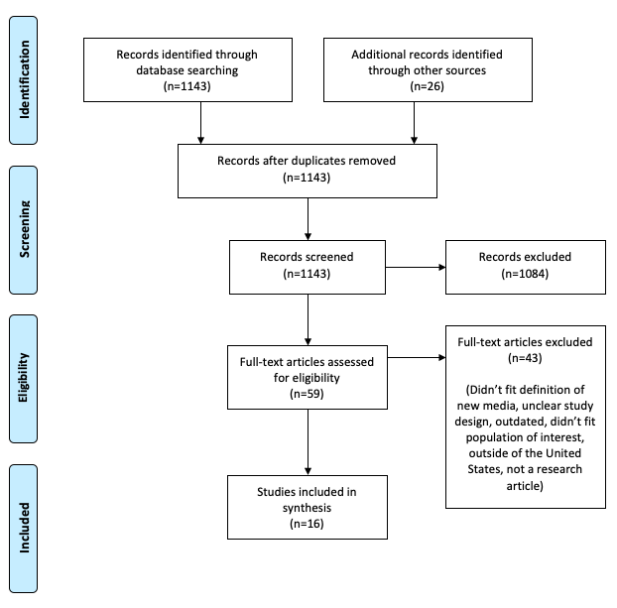
Preferred Reporting Items for Systematic Reviews and Meta-Analyses (PRISMA) diagram.

In total, 16 selected studies [15,26-40] met the inclusion criteria (see PRISMA diagram; Figure 1). Of these, 10 studies used quantitative methods [27,28,30,33,35-40], 5 studies used qualitative methods [15,26,29,31,32], and 1 study used mixed methods [34]. Reviews included in the synthesis were categorized as quantitative, qualitative, or mixed methods based on the numerical or observational nature of the studies included. The 16 included studies were summarized by the study method, type of new media platform, and sample. The most common forms of new media utilized within the included studies were social media (eg, Facebook, Twitter) [15,26-29,33-35,37,40], internet-based interventions (eg, *It’s Your Game-*Tech, *Keep It Up!*) [28,30,32,33,36], mobile applications [29,31,33,39], and interactive video games [15,38]. Studies reported various reasons for utilizing new media, including improving contraception or condom use, communicating credible information regarding HIV and STIs, reducing the transmission of HIV and STIs, improving attitudes around sexual health, and promoting STI testing–related behaviors. Around half of the studies (7/16, 44%) indicated utilizing new media as an effective sexual health promotion tool due to its ease of use and wide accessibility among adolescents and young adults [15,26,30-32,34,39].

### Synthesis of Results Table

Table 3 shows the resulting articles by the level of evidence, sample size, type of new media used, results, and any limitations within the study.

**Table 3:** Synthesis of results.

First author, year	Study method	New media platform	Sample
Bull, 2012 [27]	Cluster randomized controlled trial (RCT)	Facebook	1578 youth, 16-25 years old; 35% AA^a^
Condran, 2017 [35]	Scoping review	Social media (eg, Facebook, Twitter, Instagram)	24 articles, some AA youth
Cordova, 2015 [31]	Qualitative interviews	mHealth^b^ app	29 adolescents, 13-18 years old; 65% AA
Cordova, 2017 [36]	Systematic review	Internet	3 articles; >75% AA or Hispanic youth, 13-25 years old
Dolcini, 2015 [32]	Qualitative investigation	Internet	81 AA youth, 15-17 years old
Eason, 2017 [37]	Cross-sectional study	Facebook	112 AA adults, 18-49 years old
Fiellin, 2017 [38]	RCT	Interactive video game	333 youth, 11-14 years old; 88.6% racial/ethnic minorities
Guilamo-Ramos, 2014 [14]	Focus groups	Social networking sites, games, mobile phones	106 youth, 12-19 years old; 53% AA
Guse, 2012 [28]	Systematic review	Web-based, social networking sites	10 articles; youth 13-24 years old, some AA
Jemmott, 2017 [39]	Pilot study	iPad app	4 AA men, 18-24 years old
Muessig, 2015 [33]	Systematic review	Web-based, social media, smartphone apps	61 articles; some AA youth, 15-25 years old
Shegog, 2014 [30]	Feasibility study	Web-based	33 youth, 12-14 years old; 70% AA
Stevens, 2017 [40]	Cross-sectional study	Social media (eg, Facebook, Instagram, Twitter)	249 AA and Latino youth, 13-24 years old
Taggart, 2015 [34]	Systematic review	Social media	35 articles; 18-40 years old; some AA
Veinot, 2011 [26]	Cohort study	Social networking sites	94 youth, 14-24 years old; 80% AA
Veinot, 2013 [29]	Quasiexperimental study	Social media apps	75 AA youth, 14-24 years old

^a^AA: African American.

^b^mHealth: mobile health.

### Feasibility of New Media for Reaching Youth of Color and At-Risk Youth

Several studies cited the ability of new media to reach minority and other at-risk youth regarding sexual health [27,28,34,37]. In one study, retention of participants declined in the long term, but a significant number (1195/1578 participants, 75%) returned to complete a follow-up in the short term [27]. Still, this intervention was successful in recruiting 1578 youth, 773 of whom were minority youth [27]. Additionally, the intervention reached large numbers of youth with STI-related and HIV-related information via Facebook, with most participants viewing the content that they were intended to view; during the study period, there were 277 posts by visitors to the Just/Us Facebook page, and 93 individuals, which represents 10% of those enrolled in the intervention, were identified as “loyal” visitors to the page [27]. Another study revealed targeted Facebook messaging to be effective in reaching young African Americans living in the southeastern United States; 149 of the 176 individuals (85%) who responded to the invitation to participate in the study did so after viewing the Facebook message [37]. In a systematic review analyzing studies that used a wide range of new media (eg, social networking sites and internet-based interventions) for sexual health education, several at-risk populations were recruited, including low-income urban youth, HIV-positive youth, and minority youth [28]. Of the 10 articles included in the review, 6 articles succeeded in recruiting and retaining African American youth to a number of new media–based interventions (eg, +CLICK and MySpace) [28]. Another systematic review illustrated the capacity of new media to engage users spanning various geographic locations, ages, genders, races, and socioeconomic status when communicating about HIV; the capacity was largely due to the ability of users to remain anonymous [34]. The common feature of social media platforms that allows for the anonymity of users may encourage marginalized groups to feel more comfortable when it comes to engaging in HIV communication via such platforms [34]. Users in 6 studies reported that social media anonymity allowed for a decrease in stigma, fear, and discrimination surrounding HIV and therefore allowed participants to engage more in a discussion through social media than they would through in-person interactions [34]. This review also highlighted the usefulness of utilizing new media as a strategy for increasing access to HIV care or prevention for those who may typically face barriers to achieving such access in person, such as marginalized and at-risk groups [34].

### Ability of New Media to Change Sexual Health–Related Attitudes and Behaviors

Approximately one-third (5/16, 31%) of the studies explored the effectiveness of new media in changing attitudes and behaviors related to sexual health [30,35,37,38,40]. When comparing current practices to pre-social media service utilization, one scoping review found an increase in the utilization of services, such as an increase in the number of referrals and testing rates when social media was used as a way of reaching populations [35]. In another study, 60 of 112 participants (54%) cited viewing HIV and STI prevention messages on Facebook in the past year as the most critical factor in their decision to change their high-risk sexual behaviors [37]. In yet another study, participants improved their sexual health attitudes and knowledge 12 months after exposure to an interactive video game intervention [38]. Middle-school students who utilized an interactive, internet-based sexual health curriculum demonstrated increased perceptions of friends’ positive beliefs about delaying sex, more significant reasons for not having sex, increased self-efficacy for condom use, and greater intentions to abstain from sex until marriage [30]. The final study revealed that youth exposed to sexual health messages through social media were nearly 2.5 times more likely than youth who were not exposed to sexual health messages through social media to have used contraception or a condom at last intercourse. In contrast, parents, schools, and traditional media as information sources were not significantly related to contraception and condom use [40].

### Role of New Media in Filling Gaps in Knowledge and Information

Just under half (7/16, 44%) of the selected articles discussed the value of new media in providing information and filling critical gaps in knowledge related to sexual and reproductive health (eg, STI testing and disease prevention and management) [15,26,29,31,34,38,40]. Participants in one study shared the importance of utilizing the app as a tool for disseminating culturally specific HIV and STI information (eg, symptoms and condom use) [36]. In another study, youth who participated in an interactive video game demonstrated an average increase of 1.13 points in sexual health knowledge scores compared to the scores of youth in the control group [38]. An additional study revealed that adolescents are motivated to seek sexual health information through new media due to its accessibility and widespread use, while acknowledging in-person interactions as a frequently used resource regarding specific and reliable information [15]. To address the potential loss of trustworthiness experienced with in-person health education, new media–based interventions can utilize components of in-person interactions (eg, interactivity and specificity) [15]. Another article revealed that social media was the fourth most commonly used source of sexual risk–reduction information among African American and Latino youth, following television and movies, school, and parents [40]. However, youth in this study received information via online spaces at similar levels to information via friends and parents [40]. In one systematic review, the most common benefit of utilizing social media as a tool for HIV communication was the ability to both share and receive information [34]. Social media users also cited additional benefits of this platform: the ability to receive information regarding disease management and the ability to access nontraditional sources of information regarding HIV prevention and testing [34]. Youth who participated in focus groups suggested the use of mobile platforms and applications to address the need for credible information regarding sexual health [26]. However, participants in another study reported inconsistencies in accessing credible HIV-related and STI-related information through information technologies (eg, the internet and mobile phones), resulting in a critical gap in knowledge related to STIs [29]. Youth revealed that their most desired feature of a new media–based intervention was credible information, such as articles or question-and-answer services [29].

## Discussion

### Principal Findings

This scoping review yielded 16 distinct new media interventions to improve condom use and attitudes around sexual health, communicate credible information regarding HIV and STIs, and promote STI testing behaviors among African American youth. These interventions are distinct in their methods of new media delivery, the outcomes assessed, and their regional locations. However, they hold a shared desire to increase the overall sexual health of African American youth — a doubly vulnerable population at risk for STIs.

Several themes were identified in this review. First, new media has been shown to be a feasible method of delivery for future sexual health interventions. New media interventions received upwards of 75% participation by the African American youth who were targeted for inclusion in the studies. This is higher than the 10%-20% recruitment rate of minorities that is often the reality for the bulk of existing research [41]. One of the reasons cited for high use of new media was anonymity. The desire to conceal their identity may be of particular importance to adolescents who may not want their parents or friends to know that they are engaged in a sexual health education program. Anonymity has been found to increase participation in new media sexual health interventions among other at-risk groups, including adolescents and emerging adults [42] and adolescent women of color [43]. The decreased chances of undesired participant identification, in addition to convenience (ie, no travel requirements or synchronous login requirements) may make new media a better delivery mechanism for sexual health interventions than traditional face-to-face intervention delivery methods for some at-risk adolescents.

Next, several new media interventions identified in this scoping review were effective in changing negative attitudes and behaviors related to sexual health to more positive ones. Attitudes are an important aspect of behavior, as highlighted by the Theory of Reasoned Action (TRA) [44] and Theory of Planned Behavior (TPB) [45]. These theories purport that beliefs influence attitudes; attitudes, in turn, influence intentions; and finally, intentions influence behaviors. Understanding the link between these constructs is an important step in acknowledging the importance of attitude change as a key precursor to behavior change. TRA and TPB have been commonly used as the theoretical underpinnings for behavior change interventions. Two of the 13 interventions for African Americans that are endorsed in the “Compendium of Evidence-Based Interventions and Best Practices for HIV Prevention” use this theory in their theoretical frameworks [20].

Several new media interventions were also effective in increasing knowledge related to sexual risk reduction and helped to fill information gaps. While it is widely accepted that an increase in knowledge alone does not directly lead to risk reduction or behavior change, this construct is still often utilized by interventionists and health care researchers. It is a major component of Social Cognitive Theory (SCT) and can be applied specifically to health promotion interventions [46]. Increased knowledge may lead to better decision making and healthier behaviors. Thus, the increased knowledge reported by participants of the selected sexual health new media interventions is a positive outcome that may lead to positive changes in their sexual health behaviors. SCT is even more widely used in sexual health interventions than TRA and TPB. Eight of the 13 “Compendium of Evidence-Based Interventions and Best Practices for HIV Prevention” interventions tailored to African American youth and emerging adults utilize SCT as a theoretical underpinning [20].

While new media interventions may decrease risky sexual behavior and improve important aspects of future behavior change, comparison of new media efficacy is difficult. Any attempt to make this type of comparison may be difficult due to the wide variation of core components utilized by each intervention. Studies comparing one sexual health intervention that was implemented using various new media and traditional delivery mechanisms found no difference in outcomes [47,48]. This suggests that mode of delivery does not affect intervention efficacy, but rather, it is a mechanism used to reach a target population. It is the content of an intervention that determines its effectiveness and not its method of delivery. Other researchers have also found that the content of sexual health interventions is more indicative of effectiveness than mode of delivery [19]. Further research on the efficacy of intervention mode of delivery is warranted.

The findings of this scoping review support the use of new media platforms such as social networking sites, microblogs, online video sites, and mobile phone applications to promote safer sex behaviors and STI prevention. New media platforms should be leveraged to promote engagement in health care and reduce risk-related behaviors among African American youth. This population already utilizes new media at high rates, an indication that these platforms are highly accepted modes of communication and information delivery within this group. Moreover, African American youth have reported increased utilization of health care resources after seeing information about these services online. This suggests that new media is not a substitution for in-person services but that it can be used as a conduit for increased utilization of traditionally delivered services.

It is to the advantage of sexual health educators and interventionists to become familiar with and employ platforms that are already in heavy use by their target populations. Health care promotion and disease prevention organizations have encouraged the use of new media for the past decade [49,50]. One important factor in the creation of new media interventions is the use of adaptation methods. Instead of creating entirely new interventions, it is highly suggested to adapt evidence-based interventions for use among new populations and via new delivery methods using models such as Intervention Mapping [51], the CDC’s Map of Adaptation Process [52], and the ADAPT-ITT model [53].

### Limitations

Several limitations exist for this research. The primary limitation is the use of a broad range of platforms and measures among the studies selected for review. Furthermore, some studies utilized social media platforms, while others utilized web-based gaming platforms. Some studies measured attitudes, while others measured different sexual behaviors such as delayed onset of first sexual encounter or consistent condom use during sexual intercourse. Just as online platforms are similar yet can vary greatly in their functionality and usability, behavioral outcomes may be alike or associated in some ways without being proxies for one another; thus, they should not be compared. For this reason, it is not appropriate to attempt head-to-head comparisons of the new media interventions identified in this study. The varying factors and outcomes assessed in each intervention make it difficult to draw definitive conclusions regarding the relationship between the new media interventions and individual sexual health outcomes or behaviors. Furthermore, only a limited number of new media studies have focused solely on African American youth and young adults, and few of these interventions were gender-related. While African Americans accounted for at least 13% of each study’s participants, 11 studies in this review included other populations. Only one study was gender-specific. Therefore, while being culturally appropriate and gender-specific are highly recommended aspects of intervention creation [54-56], they may not have been important factors in many of the interventions included in this review. Finally, it was not in the scope of this study to compare the effectiveness of sexual risk-reduction interventions utilizing traditional delivery methods and those using new media. While it is not highly anticipated that intervention mode of delivery affects behavioral outcomes, this potential impact should be studied further in the presence of all intervention core components.

### Conclusions

While research in this area is limited, the results of this scoping review indicate that new media is a promising sexual health promotion tool for African American adolescents and young adults. A range of new media platforms was shown to be effective in reaching African American youth, improving sexual health–related attitudes and behaviors and filling gaps in sexual health–related knowledge and information. The encouraging results of this review suggest more research should be devoted to further exploring the benefits of utilizing new media as a tool for improving the sexual health of African American adolescents and young adults. Additionally, this review supports the value of tailoring both new and existing new media–based interventions towards this specific population.

## Abbreviations

AA: African American
AYA: adolescents and young adults
CDC: Centers for Disease Control and Prevention
mHealth: mobile health
PRISMA: Preferred Reporting Items for Systematic Reviews and Meta-Analyses
SCT: Social Cognitive Theory
SNS: social networking site
STI: sexually transmitted disease
TPB: Theory of Planned Behavior
TRA: Theory of Reasoned Action
